# *Anaerococcoides* *asporogena* gen. nov., sp. nov., a Strictly Anaerobic Bacterium, Isolated from the Dehydrated Sludge of a Steel Factory’s Wastewater Treatment Plant

**DOI:** 10.3390/microorganisms14051066

**Published:** 2026-05-09

**Authors:** Wanling Qiu, Yen-Chi Wu, Fuying Li, Yin Li, Jingjing Zhao, Shu-Jung Lai, Wangchuan Xiao, Chih-Hung Wu, Guowen Dong, Wei-Ling Zhang, Chao-Jen Shih, Sheng-Chung Chen, Hangying Zhang, Song Wang, Lintao Wu

**Affiliations:** 1 College of Environment and Safety Engineering, Fuzhou University, Fuzhou 350108, China; wanning084020@163.com (W.Q.); lijiang413508@126.com (Y.L.); zhaojj191scus@163.com (J.Z.); xwc@fjsmu.edu.cn (W.X.); chihhung@yeah.net (C.-H.W.); gwdong2008@163.com (G.D.); 2 School of Resources and Chemical Engineering, Sanming University, Sanming 365004, China; afu198207@163.com (F.L.); 18005985018@163.com (H.Z.); 19154084327@163.com (S.W.); lintaowi@163.com (L.W.); 3 Bioresource Collection and Research Center, Food Industry Research and Development Institute, Hsinchu 300193, Taiwan, China; ycw@firdi.org.tw (Y.-C.W.); zighpt@gmail.com (W.-L.Z.); 4 State Key Laboratory of Photocatalysis on Energy and Environment, Fuzhou University, Fuzhou 350108, China; 5 Department of Engineering Technology Management, International College, Krirk University, Bangkok 10220, Thailand; 6 Fujian Provincial Key Laboratory of Resources and Environmental Monitoring and Sustainable Management and Utilization, Sanming University, Sanming 365004, China; 7 Medical Plant Exploitation and Utilization Engineering Research Center, Sanming University, Sanming 365004, China; 8 College of Chemistry and Materials Science, Fujian Normal University, Fuzhou 350108, China; 9 Graduate Institute of Biomedical Sciences, China Medical University, Taichung 406040, Taiwan, China; sjlai01@gmail.com; 10 Research Center for Cancer Biology, China Medical University, Taichung 406040, Taiwan, China; 11 College of Resources and Environment, Fujian Agriculture and Forestry University, Fuzhou 350108, China

**Keywords:** sewage sludge, novel genus, *Clostridiaceae*, *Youngiibacter*, *Proteiniclasticum*, *Anaerococcoides*

## Abstract

A microbial community study using a culture-dependent method was conducted on dehydrated sludge collected from a steel factory’s wastewater treatment plant. One isolate, designated QWL-01^T^, was a strictly anaerobic, Gram-stain-negative, non-motile, non-spore-forming bacterium with coccoid cells measuring 0.6–0.9 μm in diameter. The growth of strain QWL-01^T^ was observed at 4–40 °C (optimum at 28–35 °C), pH 5.5–8.0 (optimum at pH 7.1), and a range of 0–3% NaCl (optimum at 0.5%). An analysis of the Biolog AN plate revealed positive carbon source utilization only for palatinose, α-ketovaleric acid, and pyruvic acid. The predominant fatty acids were iso-C_13:0_ (17.0%), C_16:0_ dimethyl acetal (12.0%), and anteiso-C_13:0_ (9.2%). A 16S rRNA gene sequence analysis through BLASTN demonstrated that the nearest phylogenetic neighbors of the novel strain were *Youngiibacter multivorans* DSM 6139^T^ (93.82%) and *Proteiniclasticum ruminis* JCM 14817^T^ (93.75%). The genome size of strain QWL-01^T^ was 3.69 Mbp, with a G+C content of 50.8 mol%. Comparing strain QWL-01^T^ with closely related species of genera *Proteiniclasticum* and *Youngiibacter*, the digital DNA-DNA hybridization (dDDH), average nucleotide identity (ANI), and average amino acid identity (AAI) values ranged from 26.60% to 36.80%, 65.89% to 68.30%, and 49.27% to 51.58%, respectively. Based on phenotypic, physiological, phylogenetic, and genomic relatedness evidence, strain QWL-01^T^ represents a novel genus in the family *Clostridiaceae*, for which the name *Anaerococcoides asporogena* gen. nov. sp. nov. is proposed. Strain QWL-01^T^ (=BCRC 81396^T^ = CICC 25258^T^ = NBRC 117088^T^) is the type strain of the proposed novel species.

## 1. Introduction

Sewage sludge, a byproduct of wastewater treatment plants (WWTPs), harbors a complex microbial community that plays a crucial role in the degradation of organic matter and the removal of pollutants. Despite the importance of these microbial communities, a significant portion of the microorganisms in sewage sludge remains uncultured and uncharacterized, often referred to as “microbial dark matter” (MDM) [1]. These uncultured microbes are believed to possess unique metabolic capabilities and ecological functions that are essential for the efficient operation of WWTPs, yet their roles remain largely unexplored due to the challenges associated with their cultivation and genomic characterization [2].

Traditional cultivation methods have been limited by the fact that only a small fraction of microbial species can be grown in laboratory conditions, leaving the majority of microbial diversity in sewage sludge unstudied [3]. Recent advances in metagenomics and single-cell sequencing have provided new avenues for exploring these uncultured microbes, revealing a wealth of genetic information that was previously inaccessible [4]. However, even with these technologies, the proportion of sequenced genomes from sewage sludge remains relatively low compared to other environments, such as animal-associated ecosystems [5].

Our recent study demonstrated the effectiveness of a small-scale culturomics approach for recovering previously overlooked anaerobic bacteria from complex microbiomes [6]. By combining near-full-length 16S rRNA gene amplicon sequencing with a cultivation strategy using 20 anaerobic media, four culture conditions differing in carbon source and salinity, prolonged anaerobic enrichment, and subsequent isolation by serial dilution and rolling-tube purification, we recovered 226 strictly anaerobic isolates from Styrofoam-fed *Tenebrio molitor* larvae. These isolates represented three phyla, seven classes, nine orders, 17 families, and 29 genera, including 42 known species and 34 potential novel species. Notably, 24 genera recovered by culturomics were not detected by amplicon sequencing alone, highlighting the power of this approach to expand the diversity and number of culturable anaerobes beyond those revealed by sequence-based surveys. These findings indicate that small-scale culturomics provides a useful framework for accessing rare, slow-growing, and previously uncultured anaerobic microorganisms in complex ecosystems.

In modern prokaryotic taxonomy, species- and genus-level assignments are evaluated using a polyphasic framework that integrates 16S rRNA gene phylogeny with genome-based relatedness indices. At the species level, a 16S rRNA gene sequence similarity of approximately 98.65% has been proposed as a practical reference threshold, whereas ANI values of 95–96% and dDDH values of 70% are widely accepted as genomic criteria for species delineation [7,8,9]. At the genus level, a 16S rRNA gene sequence similarity of about 94.5% is commonly used as a practical boundary, while AAI values of approximately 60–80% are generally regarded as an approximate genus-level range [10,11,12]. However, these genomic criteria should be interpreted together with phylogenomic, phenotypic, and chemotaxonomic evidence rather than as strict standalone cutoffs.

In the present study, we applied this small-scale culturomics strategy to sewage sludge collected from the wastewater treatment plant of Sanming Steel Co. Ltd. (Sanming, China) using 30 anaerobic media originally designed for methanogens, and recovered strain QWL-01^T^ as one of the novel isolates from dehydrated sludge [13]. Strain QWL-01^T^ was selected for further study because a preliminary 16S rRNA gene analysis indicated low similarity to validly published taxa, suggesting that it may represent a novel anaerobic lineage recovered from an engineered sludge environment. On the basis of phenotypic, phylogenetic, and genome-based analyses, we propose that strain QWL-01^T^ represents a novel genus and species within the family *Clostridiaceae*.

## 2. Materials and Methods

### 2.1. Anaerobic Medium Preparation

Anaerobic and modified DSM 120 media were prepared under an O_2_-free N_2_/CO_2_ (80:20, *v*/*v*) atmosphere according to the methods described previously [14,15,16,17]. The composition of 1 L modified DSM 120 medium was: K_2_HPO_4_, 0.35 g; KH_2_PO_4_, 0.23 g; NH_4_Cl, 0.50 g; MgSO_4_·7H_2_O, 0.50 g; CaCl_2_·2H_2_O, 0.25 g; cysteine hydrochloride, 0.3 g; NaHCO_3_, 2.0 g; yeast extract, 2 g; tryptone, 2 g; Na_2_S·9H_2_O, 0.3 g and 0.1% (*w*/*v*) resazurin, 0.5 mL. Solutions of vitamin [18] and trace elements [19], supplemented with sodium tungstate (Na_2_WO_4_, 0.3 mg L^−1^), were each added into the medium at a final concentration of 1% (*v*/*v*). All other components, except NaHCO_3_, yeast extract, tryptone and vitamin solution, were dissolved in boiling water. Subsequently, the excluded chemicals were added after the solution cooled down. The medium was then prepared and distributed into serum bottles or Hungate tubes with an oxygen-free atmosphere composed of N_2_/CO_2_ (80:20). These anaerobic containers were sealed and autoclaved at 121 °C for 20 min. Sodium sulfide from a sterilized anoxic stock solution was added to a final concentration of 1.0 mM for culture preservation, activation and tests of optimum growth conditions. Solid modified DSM 120 media for the roll-tube method were prepared by adding 2% (*w*/*v*) agarose to the modified DSM 120 media.

### 2.2. Sample Source and Strain Isolation

Strain QWL-01^T^ was isolated from the sewage sludge of the WWTP of Sanming Steel Co., Ltd., Fujian, China (26°14′ N, 117°36′ E). The dehydrated sewage sludge was collected on 25 June 2021 (Figure S1). Approximately 2 kg of sludge was transported to the laboratory in a plastic bag within 2 h of sampling, and about 3 mL of sludge, collected using a syringe, was inoculated into anaerobic modified DSM 120 medium and incubated at room temperature (~25 °C) for 2 weeks. Strain QWL-01^T^ was further purified and identified through three rounds of serial dilution, the rolling-tube technique [17], and 16S rRNA gene clone sequencing with primers 8F (5′-AGAGTTTGATCCTGGCTCAG-3′) and 1492RU (5′-TTTTAATTAAGGTTACGACTT-3′) [20]. Serial dilution was performed before each round of rolling-tube purification to obtain well-isolated colonies. After each round, 16S rRNA gene sequencing was used to confirm the recovery of the same bacterial strain, and purity was assessed before proceeding to the next purification round. The final purity of strain QWL-01^T^ was further supported by morphology observation and genome sequencing.

### 2.3. Morphology Observation

The morphology of strain QWL-01^T^ was examined under a phase-contrast microscope (Eclipse E600, Nikon, Tokyo, Japan), transmission electron microscopes (HT7800, Hitachi, Tokyo, Japan) with negative stain preparation [21] and a scanning electron microscope (Regulus8100, Hitachi, Tokyo, Japan) with gold sputter-coated cells [22]. Gram staining was performed according to the manufacturer’s instructions for the Gram Stain Solution Kit (Hunan BKMAN Holding Co., Ltd., Changsha, China). In addition, the 3% (*w*/*v*) KOH test was conducted to differentiate between monoderm and diderm cell envelope structures. To assess spore-forming ability, strain QWL-01^T^ was cultured in modified DSM 120 medium at 30 °C for 16 h, followed by incubation at 50 °C overnight to induce sporulation, and subsequently subjected to malachite green staining.

### 2.4. Physiology and Chemotaxonomy

The growth of strain QWL-01^T^ under aerobic and low-oxygen conditions was assessed using both solid and liquid media. For the solid-medium assays, the strain was inoculated onto modified DSM 120 agar plates and incubated at 30 °C either under anaerobic conditions or in ambient air to test for aerobic growth. For the liquid-medium assays, the strain was inoculated into thioglycollate (TGC) medium and incubated statically at 30 °C to establish an oxygen gradient. Growth distribution in the TGC medium was visually examined after 3–7 days of incubation to assess growth under low-oxygen conditions. Growth requirement tests were performed in modified DSM 120 medium lacking both yeast extract and tryptone, as well as in media supplemented with yeast extract or tryptone individually.

Growth temperature tests were conducted at 4 °C, 10 °C, 20 °C, 25 °C, 28 °C, 30 °C, 32 °C, 35 °C, 37 °C, 40 °C, and 45 °C in the presence of 0.25% (*w*/*v*) NaCl. The growth experiments for NaCl concentrations were set at 0%, 0.25%, 0.5%, 1.0%, 2.0%, 3.0%, and 5%. To measure the effect of pH on growth, the pH values of the media were modulated by the partial pressure of CO_2_, the concentration of NaHCO_3_ in the medium, or to achieve lower (<5.5) or higher (>8.0) pH values by the addition of HCl or NaOH, respectively. The pH values for the growth tests were conducted at 5.5, 6.0, 6.4, 7.1, 7.6, and 8.0. Bacterial cell growth was monitored by measuring the optical density of the broth medium at 600 nm. Each assessment was conducted in triplicate or quadruplicate.

The biochemical characteristics of strain QWL-01^T^ were determined using the AN MicroPlate (Biolog) kit (Biolog, Inc., Hayward, CA, USA) in accordance with the manufacturer’s instructions. The strain was incubated on a modified DSM 120 agar plate at 30 °C under anaerobic conditions. The Biolog AN inoculum preparation and incubation were carried out using pre-reduced medium and strict anaerobic handling to minimize oxygen exposure.

To determine the cellular fatty acids, a fatty acid methyl ester (FAME) analysis was performed using the MIDI Sherlock^TM^ Microbial Identification System (MIDI, Inc., Newark, DE, USA). The cultures were incubated on a modified DSM 120 agar plate at 30 °C for 96 h. FAMEs were extracted and prepared following the MIDI manufacturer’s protocol [23].

### 2.5. 16S rRNA Gene Phylogeny

The genomic DNA of strain QWL-01^T^ was extracted and purified following the general method mentioned by Jarrell et al. [24]. The 16S rRNA gene of strain QWL-01^T^ was amplified using primers 8F (5′-AGAGTTTGATCCTGGCTCAG-3′) and 1492RU (5′-TTTTAATTAAGGTTACCTTGTTACGACTT-3′) [20], and clone sequencing was performed by Sangon Biotech (Shanghai) Co., Ltd. (Shanghai, China). The bacterial 16S rRNA genes (listed in Table S1) and genomic sequences (listed in Tables S2 and S3) used in this study were obtained from the NCBI Reference Sequence Database [25,26], the GenBank Database [27] and the genome portal of the Department of Energy (DOE) Joint Genome Institute (JGI) [28,29,30,31]. The 16S rRNA similarity analysis was performed using BLASTN (29 MAY 2023) searches [32] or calculations with MEGA X [33]. Sequence alignment was performed using the Clustal W program [34]. The 16S rRNA gene phylogenetic tree was reconstructed by the Maximum-Likelihood (ML) [35], Neighbor-Joining (NJ) [36] and Minimum-Evolution (ME) [37] algorithms, using the MEGA X program with the Maximum Composite Likelihood substitution model [38].

### 2.6. Whole-Genome Characterization

The genome of strain QWL-01^T^ has been sequenced and reported previously [13]. Briefly, the genome was sequenced at the Sangon Biotech (Shanghai, China) Co., Ltd. using the DNBSEQ-T7 platform (MGI Tech Co., Ltd., Shenzhen, China) and MinION^TM^ sequencer (Oxford Nanopore Technology, Oxford, UK). DNBSEQ-T7 (13,928,896 reads) and MinION^TM^ (542,078 reads) reads were hybrid de novo assembled using NextDenovo v2.5.2 (https://github.com/Nextomics/NextDenovo, accessed on 15 February 2022). The hybrid sequencing protocol generated ~844X mean coverage of the genome. Gene predictions and annotations were performed using the NCBI Prokaryotic Genome Annotation Pipeline (PGAP) [39,40] and the genome annotation pipeline in the Joint Genome Institute’s Integrated Microbial Genomes Expert Review (IMG/M ER) system [28,29,30,31]. The prediction of the clustered regularly interspaced short palindromic repeats (CRISPRs) in the genome was performed by using CRISPRCasFinder [41]. Genome relatedness between strain QWL-01^T^ and related genome-available species listed in Table S3 was evaluated using digital DNA–DNA hybridization (dDDH), average nucleotide identity (ANI), and average amino acid identity (AAI). These analyses were performed using the Genome-to-Genome Distance Calculator (GGDC), EzBioCloud, and the enve-omics AAI calculator, respectively [8,42,43]. A phylogenomic tree was constructed based on the whole-genome sequences of strain QWL-01^T^ and related species using the Type (Strain) Genome Server (TYGS) [44]. The minimum-evolutionary tree was generated using FastME 2.1.6.1 software [45] based on the Genome Blast Distance Phylogeny (GBDP). Distances were determined through pairwise genome comparisons using the *d*_5_ formula [8]. GBDP pseudo-bootstrap support values were calculated using 100 replicates and the tree was rooted at the midpoint [46]. Default parameters were used for all bioinformatics analyses.

## 3. Results

### 3.1. Isolation of Strain QWL-01^T^

Strain QWL-01^T^ was further isolated and purified by using the rolling-tube technique, as detailed in our earlier study [13]. The purified isolate, designated as QWL-01^T^, has been deposited in the Bioresource Collection and Research Center, Taiwan as strain BCRC 81396^T^, the China Center of Industrial Culture Collection, China as strain CICC 25258^T^, and the Biological Resource Center, National Institute of Technology and Evaluation (NITE), Japan as strain NBRC 117088^T^.

### 3.2. Morphology of Strain QWL-01^T^

**Figure 1:**
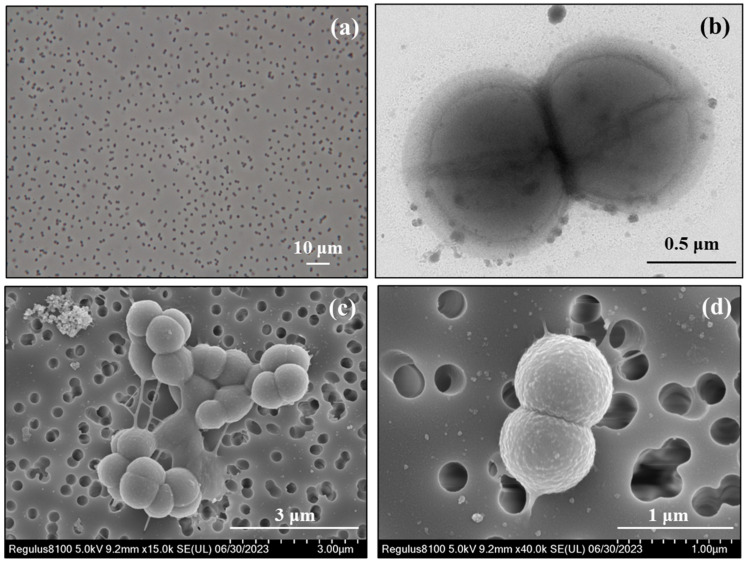
Micrographs of strain QWL-01^T^ obtained by light microscopy, transmission electron microscopy, and scanning electron microscopy. (**a**) Phase-contrast micrograph of cell morphology under Nikon Eclipse E600 microscope. (**b**) Negatively stained (2% (*w*/*v*) uranyl acetate) micrographs under transmission electron microscope (HT7800, Hitachi). (**c**,**d**) Micrograph of scanning electron microscope (Regulus8100, Hitachi). Bars: (**a**) 10 μm; (**b**) 0.5 μm; (**c**) 3 μm; (**d**) 1 μm.

The cells of strain QWL-01^T^ exhibited a coccoid morphology, measuring 0.6–0.9 μm in diameter (Figure 1a–d). Notably, cell division was observed (Figure 1b–d). The cells were non-motile and stained Gram-negative (Figure S2); however, the genome analysis indicated that strain QWL-01^T^ possesses a Gram-positive-type (monoderm) cell envelope (see Section 4.1). In addition, the cells were not lysed by 3% (*w*/*v*) KOH, supporting a monoderm, Gram-positive-type cell envelope organization and suggesting that the Gram-stain-negative result reflects atypical cell wall properties rather than a true diderm structure. Within both genera *Youngiibacter* and *Proteiniclasticum*, only *P. aestuarii* JCM 34531^T^ exhibited a coccoid morphology; all other species were rod-shaped (Table 1). No spores were observed by phase-contrast microscopy or after malachite green staining following incubation at elevated temperature (Figure S3). Consistently, the genome analysis indicated that strain QWL-01^T^ lacks the genes associated with sporulation (see Section 4.2).

### 3.3. Physiology and Chemotaxonomy of Strain QWL-01^T^

Strain QWL-01^T^ did not form colonies on DSM 120 agar plates under aerobic conditions. However, growth was observed beneath the red (oxic) layer in thioglycollate (TGC) medium, indicating tolerance to limited oxygen exposure or growth under low-oxygen conditions rather than true aerobic growth. This observation is consistent with the presence of oxygen tolerance-related genes identified in the genome of strain QWL-01^T^ (see Section 4.3). In addition, strain QWL-01^T^ did not grow in modified DSM 120 medium lacking both yeast extract and tryptone, but growth was observed when either yeast extract or tryptone was supplied individually.

The cells were able to grow at 4 °C, but very slowly. The growth temperature range of QWL-01^T^ was 4 °C to 40 °C, with an optimal growth temperature between 28 and 35 °C (Table 1, Figure S4a). Strain QWL-01^T^ grew over the NaCl concentration range of 0% to 3% (*w*/*v*) NaCl and the optimal condition was 0.5% (*w*/*v*) NaCl (Table 1, Figure S4b). Strain QWL-01^T^ was able to grow over the pH range of 5.5 to 8.0 and showed optimal growth at a pH of 7.1 (Table 1, Figure S4c). These results indicated that strain QWL-01^T^ is a mesophilic and neutrophilic bacterium.

The AN MicroPlate test indicated that positive reactions were observed only for palatinose, α-ketovaleric acid, and pyruvic acid. The major fatty acids found in strain QWL-01^T^ were iso-C_13:0_ (17.0%), C_16:0_ dimethyl acetal (12.0%), and anteiso-C_13:0_ (9.2%) along with other less abundant fatty acids C_16:1_ ω7c (6.2%), C_16:0_ (5.8%), and iso-C_17:1_ ω10c (5.7%) (Table 2). This profile was considerably different from those of its phylogenetically closest neighbors listed in Table 2.

### 3.4. 16S rRNA Gene Phylogeny of Strain QWL-01^T^ and Related Taxa

**Figure 2:**
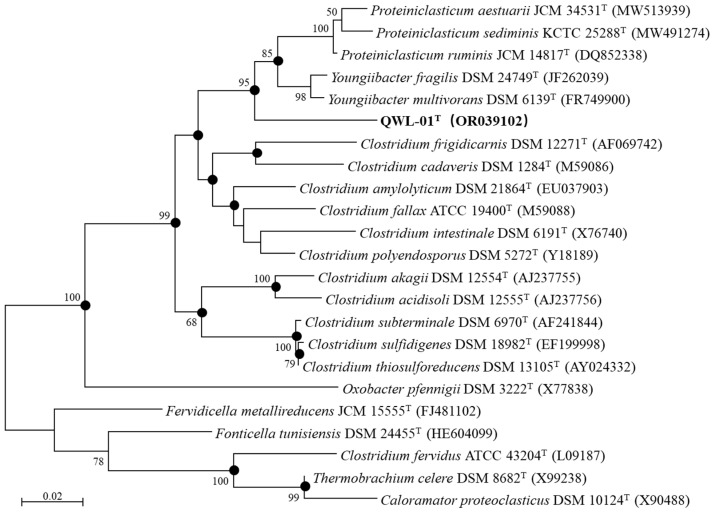
A phylogenetic analysis of the 16S rRNA gene sequences showed the relationship between strain QWL-01^T^ and the related genera. Sequence alignment was performed using the Clustal W program. This tree was performed with MEGA X software and constructed based on a subset of nearly full-length sequences by the Maximum-Likelihood method. The bootstrap values at the nodes are shown as percentages based on an ML analysis of 1000 resampled datasets. Only values > 50% are shown. The accession number for each reference species is shown in parentheses. Bar, 0.02 evolutionary distances. ●, ML, NJ and ME on one branch.

Based on the 16S rRNA gene similarity analysis using BLASTN searches, strain QWL-01^T^ was most closely related to *Youngiibacter multivorans* DSM 6139^T^ [47,48] and *Proteiniclasticum ruminis* JCM 14817^T^ [50], with similarities of 93.82% and 93.75%, respectively (93.48% and 92.74% according to MEGA X calculations; Table S1). Additionally, the 16S rRNA gene similarities between strain QWL-01^T^ and species within the genera *Youngiibacter* and *Proteiniclasticum* ranged from 93.31% to 93.48% and 92.34% to 92.89%, respectively (Table S1). A phylogenetic analysis of the 16S rRNA gene sequences (Figure 2) showed that strain QWL-01^T^ formed a distinct lineage outside the clades of both *Youngiibacter* and *Proteiniclasticum*. These results suggest that strain QWL-01^T^ may represent a novel genus within the family *Clostridiaceae*.

### 3.5. Genomic Relatedness Analyses

The assembly generated a single large contig of 3,691,162 bp with 50.81% GC content (Table 1 and Table S3). The G+C content of the genome of strain QWL-01^T^ differs significantly from that of the two *Youngiibacter* type strains (44.84–46.57%) and the three *Proteiniclasticum* type strains (43.07–51.35%), with differences of 4.24–5.97% and 0.54–7.73%, respectively (Table 1 and Table S3). The genome was annotated by the NCBI PGAP to have 3360 genes, of which 3232 were protein coding. The genome contains 15 rRNA genes and 53 tRNA genes. One CRISPR with a high evidence level was found in the genome by using CRISPRCasFinder.

To further clarify the taxonomic position of strain QWL-01^T^, genomic relatedness analyses were performed against closely related members of the genera *Youngiibacter* and *Proteiniclasticum* (Tables S2 and S3). The resulting dDDH, ANI, and AAI values were 26.60–36.80%, 65.89–68.30%, and 49.27–51.58%, respectively (Tables S4 and S5). Since dDDH and ANI are widely used for species-level delineation, the low values obtained here, far below the accepted thresholds of 70% and 95–96%, respectively [7,8,9], clearly exclude strain QWL-01^T^ from all previously described species. For genus-level assessment, AAI provides a more informative genomic metric. The AAI values between strain QWL-01^T^ and representatives of *Youngiibacter* and *Proteiniclasticum* were markedly lower than the commonly referenced genus-level range of approximately 60–80% [11,12], indicating that strain QWL-01^T^ cannot be assigned to either genus. Therefore, these genomic data support the proposal that strain QWL-01^T^ represents a novel genus rather than a novel species within *Youngiibacter* or *Proteiniclasticum*. Furthermore, TYGS-based phylogenetic analysis clearly showed that strain QWL-01^T^ formed a distinct lineage separate from the clades representing the genera *Youngiibacter* and *Proteiniclasticum* (Figure S5). Collectively, these results support the proposal that strain QWL-01^T^ represents a novel genus within the family *Clostridiaceae*. Since both the 16S rRNA gene sequence similarities and the AAI values are below the commonly accepted genus-level reference ranges, strain QWL-01^T^ is more appropriately classified as representing a novel genus rather than a novel species within either *Youngiibacter* or *Proteiniclasticum*.

## 4. Discussion

### 4.1. Gram Reaction and Cell Envelope Structure

Strain QWL-01^T^ showed a Gram-stain-negative reaction under the conditions tested. However, this observation does not necessarily contradict its phylogenetic and genomic affiliation with the family *Clostridiaceae*. In members of Bacillota (Firmicutes), particularly in some clostridial lineages, Gram staining may be variable or even negative despite the presence of a Gram-positive-type cell envelope. Such staining behavior is generally attributed to the known limitations of the Gram-staining reaction, including rapid decolorization, culture age, and cell wall changes associated with reduced viability, rather than to the presence of a canonical diderm (Gram-negative-type) cell envelope [52]. Consistent with this interpretation, the closest related genera *Youngiibacter* and *Proteiniclasticum* have also been reported to stain Gram-negative in their original descriptions [48,50]. Moreover, the genome analysis of strain QWL-01^T^ did not identify canonical genes associated with lipopolysaccharide biosynthesis or outer-membrane biogenesis/assembly, including the *lpx*, *lpt*, *msbA*, *bamA*, and *lol* systems (Table S6) [53,54]. In contrast, multiple sortase genes and several proteins carrying LPXTG-type cell wall sorting motifs were detected (Table S6), features that are characteristic of monoderm, Gram-positive-type cell envelope organization [55,56]. Taken together, these results indicate that the Gram-stain-negative reaction of strain QWL-01^T^ should be interpreted cautiously as a staining phenotype and does not contradict its genome-based taxonomic placement or its inferred monoderm cell envelope structure.

### 4.2. Sporulation

No spores were observed for strain QWL-01^T^ by phase-contrast microscopy or after malachite green staining following incubation at elevated temperature (Figure 2). In addition, the genome analysis did not identify the key genes associated with sporulation, including the master regulator *spo0A* and the genes involved in sporulation stages II–V [57]. Taken together, these phenotypic and genomic data support the conclusion that strain QWL-01^T^ is non-spore-forming.

### 4.3. Oxygen Tolerance and Genomic Basis

Although strain QWL-01^T^ did not grow under aerobic conditions, growth was observed beneath the oxic layer in TGC medium, indicating tolerance to limited oxygen exposure or the ability to persist under low-oxygen conditions rather than true aerobic growth. The genome analysis revealed the presence of multiple genes associated with oxidative stress response, including superoxide dismutase (WFF72806.1), rubrerythrin (WFF74505.1), peroxidase (WFF74103.1), NADH oxidase (WFF73171.1), and thioredoxin systems (WFF73345.1, WFF73140.1). These enzymes are generally associated with the detoxification of reactive oxygen species and the maintenance of intracellular redox balance. Therefore, the presence of the corresponding genes in the genome is consistent with a potential capacity of strain QWL-01^T^ to tolerate transient or low levels of oxygen, although this function was not directly demonstrated experimentally in the present study.

### 4.4. Predicted Fermentative Metabolism and Ecological Role of QWL-01^T^

The genome annotation of strain QWL-01^T^ suggests a predominantly fermentative anaerobic metabolism. Core glycolytic enzymes were identified, together with pyruvate:ferredoxin oxidoreductase (WFF71677), indicating the conversion of pyruvate to acetyl-CoA under anoxic conditions. Downstream fermentative pathways include genes for phosphate acetyltransferase (WFF74239) and acetate kinase (WFF74240), as well as phosphate butyryltransferase (WFF73354) and butyrate kinase (WFF73353/WFF73355), suggesting the potential production of acetate and butyrate. The presence of lactate dehydrogenase (WFF71999) further indicates a possible route to lactate formation. In addition, the genome encodes group A [FeFe] hydrogenases (WFF71763/WFF72845) and associated maturation proteins, consistent with H_2_ production during redox balancing.

QWL-01^T^ also appears adapted to the utilization of soluble organic matter in sludge. The genome encodes multiple peptidases/proteases, peptide and amino acid transport systems (WFF73363/WFF73135), and several enzymes involved in amino-acid-based fermentative metabolism, including D-proline reductase (WFF72854/WFF72856–57) and glycine/sarcosine/betaine reductase-related proteins (WFF73141–73145). These traits suggest the ability to use peptides and amino acids released from protein-rich sludge substrates. Genes related to sugar uptake and catabolism, including phosphotransferase system components (WFF71510/WFF72078) and enzymes involved in the metabolism of glucose and N-acetylglucosamine (WFF73216/WFF73888/WFF72811/WFF72081), indicate that simple carbohydrates and amino-sugar-derived compounds may also serve as substrates.

Taken together, these features suggest that QWL-01^T^ functions as a secondary fermenter in anaerobic sludge ecosystems. It likely contributes to carbon cycling by converting hydrolysis-derived soluble organics into acetate, butyrate, lactate, H_2_, and related intermediates, which can be further consumed by syntrophic microorganisms and methanogenic archaea.

### 4.5. Putative Detoxification and Pollutant Transformation Potential of Strain QWL-01^T^

The genome annotation suggests that strain QWL-01 possesses several detoxification and stress-response systems relevant to polluted industrial sludge, including a putative arsenate detoxification module (ArsR-ArsC-ACR3, WFF73836, WFF73838-39, WFF72140), chromate resistance transporters (WFF72179-80), multiple heavy-metal-translocating P-type ATPases (WFF71627, WFF71661, WFF72833), and redox enzymes such as nitroreductases (WFF71643, WFF73380) and flavin reductases (WFF72582, WFF74343). In addition, the presence of a putative aromatic ring-hydroxylating dioxygenase alpha subunit (WFF72647) and a nitrilase-related carbon-nitrogen hydrolase (WFF72109) suggests a limited potential for the transformation of selected aromatic or nitrogen-containing xenobiotic compounds. These predictions indicate adaptation to metal- and toxin-stressed sludge environments, although the substrate range and biodegradation capacity remain to be validated experimentally.

### 4.6. Phenotypic and Chemotaxonomic Evidence Supporting Genus Delineation

The Biolog AN results for strain QWL-01^T^ were limited, with only a few substrates showing positive reactions. This pattern likely reflects both the relatively narrow substrate spectrum of the strain and its strictly anaerobic lifestyle. In contrast to the more saccharolytic phenotype reported for *Youngiibacter* [47,48] and the proteolytic lifestyle described for *Proteiniclasticum ruminis* [50], strain QWL-01^T^ exhibited a comparatively restricted Biolog AN substrate utilization profile under the conditions tested. Nevertheless, comparison with related genera indicates that strain QWL-01^T^ differs from *Youngiibacter* and *Proteiniclasticum* in its substrate utilization pattern, supporting its phenotypic distinctiveness in conjunction with the phylogenetic and genomic evidence.

Members of *Youngiibacter* were originally described as strictly anaerobic, non-spore-forming, non-motile, rod-shaped bacteria, with major fatty acids dominated by C_16:0_ ALDE and summed feature 3 (C_16:1_ ω7c and/or C_16:1_ ω6c) [47]. *Proteiniclasticum ruminis*, the type species of *Proteiniclasticum*, was described as a strictly anaerobic, non-motile, non-spore-forming, rod-shaped and predominantly proteolytic bacterium whose fatty acid profile is characterized mainly by iso-branched fatty acids [50]; similarly, *Proteiniclasticum aestuarii* also shows major fatty acids centered on iso-C_15:0_ and anteiso-C_15:0_ [49]. In contrast, strain QWL-01^T^ exhibits a stable coccoid morphology, is non-spore-forming, shows a distinct physiological profile, and possesses a fatty acid composition dominated by iso-C_13:0_, C_16:0_ dimethyl acetal, and anteiso-C_13:0_. These differences in cell morphology, physiology, and fatty acid composition provide additional phenotypic and chemotaxonomic support for the proposal that strain QWL-01^T^ represents a novel genus.

### 4.7. Taxonomic Placement of Strain QWL-01^T^ Within Current Clostridiaceae Systematics

Recent taxonomic studies of *Clostridiaceae* and related anaerobic lineages have increasingly shown that 16S rRNA gene phylogeny alone is often insufficient for robust genus-level assignment, particularly in groups with broad historical classifications and overlapping phenotypic traits. Instead, current prokaryotic taxonomy emphasizes a genome-based polyphasic framework that integrates phylogenomics with genomic similarity indices such as ANI, dDDH, and AAI, together with phenotypic and chemotaxonomic data [58,59,60,61]. This trend is reflected in the recent update of the minimal standards for the use of genome data in prokaryotic taxonomy, as well as in recent phylogenomic re-evaluations of related anaerobic families, which have demonstrated the importance of genome-scale evidence for refining higher-level taxonomic boundaries. In this context, the taxonomic placement of strain QWL-01^T^ is consistent with current systematic practice: its distinct position is supported not only by 16S rRNA gene divergence, but also by TYGS-based genome phylogeny, low ANI/dDDH and AAI values relative to *Youngiibacter* and *Proteiniclasticum*, and clear phenotypic and fatty acid differences. Taken together, these data support the proposal that strain QWL-01^T^ represents a novel genus within the family *Clostridiaceae*.

## 5. Conclusions

Based on phenotypic, physiological, phylogenetic, and genomic relatedness evidence, strain QWL-01^T^ represents a novel genus in the family *Clostridiaceae*, for which the name *Anaerococcoides asporogena* gen. nov. sp. nov. is proposed.

**DESCRIPTION OF** ***ANAEROCOCCOIDES*** **GEN. NOV.**

*Anaerococcoides* (An.ae.ro.coc’co.i.des. Gr. pref. an-, not; Gr. n. aer, air; N.L. masc. n. coccus, a coccus; Gr. suff. -oides, resembling; N.L. neut. n. *Anaerococcoides*, a coccus-like strictly anaerobic bacterium).

The cells are strictly anaerobic, non-motile, non-spore-forming cocci. Chemoorganotrophic and fermentative. The predominant cellular fatty acids are iso-C_13:0_, C_16:0_ dimethyl acetal and anteiso-C_13:0_. The genomic DNA G+C content is around 50–51 mol%. Phylogenetically affiliated with the family *Clostridiaceae*. The type species is *Anaerococcoides asporogena*.

**DESCRIPTION OF** ***ANAEROCOCCOIDES ASPOROGENA*** **SP. NOV.**

*Anaerococcoides asporogena* (a.spo.ro’ge.na. Gr. pref. a-, not; Gr. n. spora, seed, spore; N.L. neut. adj. *asporogena*, non-spore-forming).

The cells are strictly anaerobic, non-motile, Gram-stain-negative, non-spore-forming cocci, measuring 0.6–0.9 μm in diameter. Growth occurs in modified DSM 120 medium at pH 5.5–8.0 (optimum pH 7.1), at temperatures ranging from 4 to 40 °C (optimum 28–35 °C), and in the presence of 0–3% (*w*/*v*) NaCl (optimum 0.5%). The genomic DNA G+C content is 50.81 mol%, as determined by whole-genome sequencing. The species was isolated from sewage sludge collected at the Wastewater Treatment Plant of Sanming Steel Co., Ltd., Fujian Province, China. The type strain is QWL-01^T^ (= BCRC 81396^T^ = CICC 25258^T^ = NBRC 117088^T^).

Beyond its taxonomic novelty, phenotypic and genome-based evidence suggests that this strain is a strictly anaerobic fermentative bacterium that may contribute to the turnover of soluble organic matter in sludge ecosystems. The presence of genes related to oxidative stress response, detoxification, and putative pollutant transformation further indicates potential adaptation to industrial sludge environments. These features suggest that strain QWL-01^T^ may be relevant not only to the ecology of anaerobic sludge communities but also as a microbial resource for future studies on anaerobic metabolism, pollutant tolerance, and biotechnology. However, these proposed functional roles remain to be validated experimentally.

## Figures and Tables

**Table 1:** Comparison of physiological characteristics of strain QWL-01^T^ and related strains of both genera *Youngiibacter* and *Proteiniclasticum*.

Characteristics\Strains	1	2	3	4	5	6
Cell morphology (μm)	cocci (0.6–0.9)	rod (0.5–0.9 × 0.8–3.5)	rod (0.4–0.5 × 1.3–2.0)	cocci (0.7–1.0)	rod (0.5–0.8 × 0.6–2.0)	rod (0.5–0.8 × 2.0–3.5)
Motility	−	+	−	+	−	−
Temp. range (°C) (optimum)	4–40 (28–35)	20–35 (30)	15–36 (30–36)	4–41 (34–37)	24–46 (38–39)	20–47 (37)
NaCl range (%, *w*/*v*) (optimum)	0–3 (0.5)	NR	0–3 (1)	0–8 (0–2)	0–5 (0–2)	0–1 (0)
Growth pH range (optimum)	5.5–8.0 (7.01)	7.2–8.6 (7.8)	6.0–8.0 (7.0–8.0)	6.5–10.0 (7.0–7.5)	5.6–8.7 (7.0–7.3)	6.0–9.0 (7.5)
Genome size (Mbp)	3.69	3.67	3.99	3.18	3.12	2.99
Genomic DNA G+C content (mol%)	50.81 (Gs)	44.84 (Gs)	46.57 (Gs)	45.57 (Gs)	43.07 (Gs)	51.35 (Gs)
Source	sewage sludge (China)	anoxic sludge-oil refinery wastewater treatment facility (China)	natural gas production-water (China)	tidal flat sediment (Suncheon Bay, South Korea)	yak rumen (China)	anaerobic sludge (China)

Strains: 1, strain QWL-01^T^; 2, *Y. multivorans* DSM 6139^T^ [47]; 3, *Y. fragilis* DSM 24749^T^ [48]; 4, *P. aestuarii* JCM 34531^T^ [49]; 5, *P. ruminis* JCM 14817^T^ [50]; 6, *P. sediminis* KCTC 25288^T^ [51]; +, positive reaction; −, negative reaction; NR, not reported; Gs, data from genome sequencing.

**Table 2:** A comparison of the fatty acid profiles of strain QWL-01^T^ and closely related strains. Strains: 1, strain QWL-01^T^ (data from this study); 2, *Y. fragilis* DSM 24749^T^ [48]; 3, *P. aestuarii* JCM 34531^T^ [49]; 4, *P. ruminis* JCM 14817^T^ [50]; 5, *P. sediminis* KCTC 25288^T^ [51]. The values are percentages of the total fatty acids. The major fatty acids (>10%) are highlighted in bold type. −, not detected or <2%. The major fatty acids reported for *Youngiibacter multivorans* DSM 6139^T^ were summed feature 3 (C16:1 ω7c and/or C16:1 ω6c) and C16:0 ALDE [47]; however, this strain was not included in Table 2 because no more detailed fatty acid composition data were available.

Fatty Acid\Strains	1	2	3	4	5
12:0	2.4	–	–	1.4	–
13:0 iso	**17.0**	–	6.0	**19.2**	1.0
13:0 anteiso	9.2	–	3.5	**10.0**	1.1
14:0 iso	1.1	2.0	2.8	**19.4**	2.3
14:0	3.5	2.0	1.6	15.1	–
14:0 2-OH	–	2.7	–	–	–
15:1 iso ω9c	3.4	–	–	–	–
15:0 iso	5.0	–	**27.2**	**15.2**	**25.1**
15:0 anteiso	2.8	–	**16.9**	9.2	**13.0**
16:0 aldehyde	2.9	**23.3**	–	–	–
15:0 iso DMA	3.0	–	–	–	7. 6
16:1 ω7c alcohol	1.3	–	–	–	–
15:1 ω5c	–	–	–	2.6	–
16:1 ω9c	–	9.5	–	–	–
16:1 ω6c	–	**28.5**	–	–	–
16:1 ω7c	6.2	–	–	1.3	–
16:1 ω5c	1.0	3.4	–	–	–
16:0	5.8	7.2	6.6	1.2	**11.6**
17:1 iso ω10c	5.7	–	–	–	–
16:0 DMA	**12.0**	–	–	–	8.1
16:0 iso	–	1.2	6.6	–	2.1
16:1 2-OH	–	–	8.6	–	–
17:0 iso	1.9	–	6.6	–	4.2
17:0 cyclo ω7c	4.5	–	–	–	–
17:0 ω7c	–	3.3	–	–	–
18:0	0.8	4.6	1.3	–	2.2

## Data Availability

The 16S rRNA gene sequence and complete genome sequence of strain QWL-01T are openly available in NCBI at https://www.ncbi.nlm.nih.gov/nuccore/OR039102.1, reference number OR039102, and https://www.ncbi.nlm.nih.gov/nuccore/CP120965.1, reference number CP120965. Accessed on 29 May 2023.

## Supplementary Materials

The following supporting information can be downloaded at https://www.mdpi.com/article/10.3390/microorganisms14051066/s1, Figure S1: A photograph of the sludge dewatering devices and dehydrated sludge collected at the wastewater treatment plant of the Sanming Steel Co. Ltd. in Sanming City, Fujian Province, China, taken on 25 June 2021; Figure S2: Gram-staining micrograph of strain QWL-01^T^. Cells were stained using the standard Gram-staining procedure and observed under a light microscope. Strain QWL-01^T^ exhibited a Gram-negative staining reaction, appearing red after counterstaining. Scale bar, 5 μm; Figure S3: Malachite green spore-staining micrographs of strain QWL-01^T^. (a) Cells cultured anaerobically at 30 °C for 3 days and then stained using the malachite green spore-staining method. (b) Cells initially cultured at 30 °C for 16 h and subsequently incubated at 50 °C for 1 day prior to malachite green staining. In both conditions, strain QWL-01^T^ exhibited no detectable endospore formation, with vegetative cells counterstained red and no green-stained spores observed. Scale bar, 10 μm; Figure S4: Influence of (a) temperature, (b) NaCl concentration and (c) pH on the growth of strain QWL-01^T^ inoculated into modified DSM 120 media. The growth temperature tests were conducted at 4 °C, 10 °C, 20 °C, 25 °C, 28 °C, 30 °C, 32 °C, 35 °C, 37 °C, 40 °C, and 45 °C in the presence of 0.25% NaCl. The growth experiment of NaCl concentrations were set at 0 %, 0.25%, 0.5%, 1%, 2%, 3%, and 5%. The pH values for growth tests were conducted at 5.5, 6.0, 6.4, 7.1, 7.6, and 8.0. The temperature was set to 30 °C for the NaCl concentration and pH growth tests. Specific growth rates were calculated based on the increase in optical density during the logarithmic growth phase and were presented as the means of triplicate or quadruplicate cultures; Figure S5: TYGS phylogenetic tree based on whole-proteome showing relationships between strain QWL-01^T^ (red) and related taxa (listed in Table S2). Tree inferred with FastME 2.1.4 from Genome BLAST Distance Phylogeny (GBDP) distances calculated from genome sequences. Branch lengths are scaled in terms of GBDP distance formula d5; numbers at the nodes are GBDP pseudo-bootstrap support values from 100 replicates. Leaf labels are annotated by affiliation to species and subspecies clusters, genomic G+C content, δ values, overall genome size, and number of proteins; Table S1: The 16S rRNA gene sequence similarity matrix between strain QWL-01^T^ and related taxa; Table S2: List of available genomes related to strain QWL-01^T^, which were used for analyses via digital DNA-DNA Hybridization (dDDH), Average Nucleotide Identity (ANI), Average Amino Acid Identity (AAI), and the Type Strain Genome Server (TYGS); Table S3: General genomic information and features of strain QWL-01^T^ and closely related taxa in the genera *Proteiniclasticum* (*Prot.*) and *Youngiibacter* (*Young.*). These detailed data were obtained from the JGI/IMG MER database.; Table S4: The pairwise analyses of dDDH (upper right part, unit: %) and OrthoANIu (lower left part, unit: %) for strain QWL-01^T^ and other related taxa; Table S5: The pairwise analyses of AAI (lower left part, unit: %) for strain QWL-01^T^ and other related taxa; Table S6: Genome-based inference of the cell envelope structure of strain QWL-01^T^.

## Abbreviations

WWTPs, wastewater treatment plants; MDM, microbial dark matter; FAME, fatty acid methyl ester; ANI, average nucleotide identity; AAI, average amino acid identity; dDDH, digital DNA-DNA hybridization; GBDP, genome blast distance phylogeny; TYGS, type (strain) genome server.
