# Supplementary material for: Anaerococcoides asporogena gen. nov., sp. nov., a Strictly Anaerobic Bacterium, Isolated from the Dehydrated Sludge of a Steel Factory’s Wastewater Treatment Plant
Source: Microorganisms. 2026 May 9;14(5):1066. doi: 10.3390/microorganisms14051066 (PMC13209922; doi:10.3390/microorganisms14051066)
Supplement: Supplementary file 1 [file microorganisms-14-01066-s001.zip › microorganisms-4265983-supplementary/Supplementary Data_QWL-01.pdf]

Supplementary Data

Anaerococcoides asporogena gen. nov., sp. nov., a strictly anaerobic bacterium, isolated from dehydrated sludge of a steel factory's waste water treatment plant

Wanling Qiu[1,2†], Yen-Chi Wu[3†], Fuying Li[2,4,5,6], Yin Li[1,2,6,7], Jingjing Zhao[1,2,6,7,8], Shu-Jung Lai[9,10], Wangchuan Xiao[1,2,6,7], Chih-Hung Wu[1,2,6,7], Guowen Dong[1,2,6,7,11], Yi-Ting You[2], Wei-Ling Zhang[3], Chao-Jen Shih[3*], Sheng-Chung Chen[1,2,6,7*], Hangying Zhang[2,6,7], Song Wang[2,6,7,8], Lintao Wu[2,6,7,11]

[1]College of Environment and Safety Engineering, Fuzhou University, Fuzhou, Fujian, People's Republic of China

[2]School of Resources and Chemical Engineering, Sanming University, Sanming City, Fujian, People's Republic of China

[3]Bioresource Collection and Research Center, Food Industry Research and Development Institute, Hsinchu, Taiwan, Republic of China

[4]State Key Laboratory of Photocatalysis on Energy and Environment, Fuzhou University, Fuzhou, Fujian, People's Republic of China

[5]Department of Engineering Technology Management, International College, Krirk University, Bangkok 10220, Thailand

[6]Fujian Provincial Key Laboratory of Resources and Environmental Monitoring and Sustainable Management and Utilization, Sanming University, Sanming, Fujian, People's Republic of China

[7]Medical Plant Exploitation and Utilization Engineering Research Center, Sanming

University, Sanming, Fujian, People's Republic of China

[8]College of Chemistry and Materials Science, Fujian Normal University, Fuzhou, Fujian, People's Republic of China

[9]Graduate Institute of Biomedical Sciences, China Medical University, Taichung City, Taiwan, Republic of China

[10]Research Center for Cancer Biology, China Medical University, Taichung City, Taiwan, Republic of China

[11]College of Resources and Environment, Fujian Agriculture and Forestry University, Fuzhou, Fujian, People's Republic of China

Subject category: New Taxa-Bacteria; Bacillota

***Corresponding author:** CJS, cjs23@firdi.org.tw; SCC, benbear.xe@gmail.com

[†]These authors contributed equally to this work.

6 Supplementary Tables

5 Supplementary Figures

Table S1. The 16S rRNA gene sequence similarity matrix between strain QWL-01^{T} and related taxa.

[a]Strains: **1. QWL-01^{T}**; 2. *Youngiibacter multivorans* DSM 6139^{T}; 3. *Youngiibacter fragilis* DSM 24749^{T}; 4. *Proteiniclasticum aestuarii* JCM 34531^{T}; 5. *Proteiniclasticum ruminis* JCM 14817^{T}; 6. *Proteiniclasticum sediminis* KCTC 25288^{T}; 7. *Clostridium polyendosporum* DSM 5272^{T}; 8. *Clostridium amylolyticum* DSM 21864^{T}; 9. *Clostridium fallax* ATCC 19400^{T}; 10. *Clostridium cadaveris* DSM 1284^{T}; 11. *Clostridium akagii* DSM 12554^{T}; 12. *Clostridium intestinale* DSM 6191^{T}; 13. *Clostridium frigidicarnis* DSM 12271^{T}; 14. *Clostridium acidisoli* DSM 12555^{T}; 15. *Clostridium subterminale* DSM 6970^{T}; 16. *Clostridium sulfidigenes* DSM 18982^{T}; 17. *Clostridium thiosulforeducens* DSM 13105^{T}; 18. *Fervidicella metallireducens* JCM 15555^{T}; 19. *Oxobacter pfennigii* DSM 3222^{T}; 20. *Fonticella tunisiensis* DSM 24455^{T}; 21. *Thermobrachium celere* DSM 8682^{T}; 22. *Caloramator fervidus* ATCC 43204^{T}; 23. *Caloramator proteoclasticus* DSM 10124^{T}. [b]Similarities were calculated by MEGA X.

	**1[a]**	**2**	**3**	**4**	**5**	**6**	**7**	**8**	**9**	**10**	**11**	**12**	**13**	**14**	**15**	**16**	**17**	**18**	**19**	**20**	**21**	**22**	**23**
**1**	**100[b]**																						
**2**	**93.48**	100																					
**3**	**93.31**	98.45	100																				
**4**	**92.89**	95.61	94.73	100																			
**5**	**92.74**	95.32	94.88	98.19	100																		
**6**	**92.34**	95.25	95.02	97.58	97.45	100																	
**7**	**92.10**	92.67	92.28	92.89	92.77	92.52	100																
**8**	**91.53**	92.76	91.97	91.83	92.20	91.53	95.34	100															
**9**	**90.96**	91.29	91.19	91.02	90.65	90.62	94.97	95.44	100														
**10**	**90.82**	92.13	92.09	91.09	90.77	90.66	92.07	92.63	91.99	100													
**11**	**90.72**	92.62	92.03	93.01	92.60	92.44	92.08	93.62	91.79	91.19	100												
**12**	**90.07**	91.20	90.89	90.28	90.07	89.46	94.34	93.98	93.92	91.05	90.86	100											
**13**	**89.15**	90.17	89.94	89.44	89.59	89.41	92.56	94.79	93.71	92.98	91.71	92.39	100										
**14**	**89.62**	91.35	90.91	91.58	91.50	91.20	91.84	93.05	91.39	90.17	97.33	90.53	90.94	100									
**15**	**89.31**	91.70	91.65	91.28	90.86	90.57	92.30	92.33	91.27	91.40	93.07	91.07	90.88	93.16	100								
**16**	**89.54**	91.62	91.56	91.10	91.07	90.57	92.14	92.69	91.49	91.39	93.14	91.06	91.03	93.45	98.93	100							
**17**	**89.73**	91.57	91.53	91.20	90.79	90.65	92.14	92.64	91.61	91.59	93.06	91.15	90.99	93.35	99.58	99.58	100						
**18**	**87.00**	87.19	87.41	86.98	87.50	87.06	88.11	88.10	86.87	86.58	87.24	86.67	86.56	87.22	86.72	86.87	86.29	100					
**19**	**85.26**	86.94	87.10	86.53	86.61	87.16	86.03	86.05	86.05	85.04	85.06	86.20	84.69	85.14	85.62	85.94	86.38	85.64	100				
**20**	**84.87**	85.68	85.44	85.39	86.43	85.79	86.97	87.60	85.63	85.09	86.71	85.16	86.14	86.66	86.11	86.60	85.66	91.93	84.55	100			
**21**	**83.46**	85.25	85.68	85.13	85.63	85.83	86.41	86.27	84.87	84.01	85.26	84.85	85.63	85.34	85.38	85.54	84.98	89.36	86.49	90.77	100		
**22**	**83.23**	84.32	84.31	83.99	84.89	84.28	85.09	84.91	83.61	82.22	83.97	83.16	83.48	84.02	84.03	84.61	83.68	88.33	84.89	89.53	94.29	100	
**23**	**82.12**	83.83	84.03	83.14	82.31	83.46	83.96	83.70	82.21	81.70	83.15	82.69	82.97	83.06	82.67	82.83	82.66	87.68	84.50	87.81	97.59	91.43	100

Table S2. List of available genomes related to strain QWL-01^{T}, which were used for analyses via digital DNA-DNA Hybridization (dDDH), Average Nucleotide Identity (ANI), Average Amino Acid Identity (AAI), and the Type Strain Genome Server (TYGS).

No.	Species	GenBank accession number
**01**	**QWL-01^{T}**	**CP120965**
**02**	*Proteiniclasticum aestuarii* SCR006^{T}	JAFNJU000000000
**03**	*Proteiniclasticum sediminis* BAD-10^{T}	JAGSCS000000000
**04**	*Proteiniclasticum ruminis* DSM 24773^{T}	JNKC00000000
**05**	*Youngiibacter fragilis* 232.1^{T}	AXUN00000000
**06**	*Youngiibacter multivorans* DSM 6139^{T}	JAGGKC000000000
**07**	*Clostridium frigidicarnis* DSM 12271^{T}	FOKI00000000
**08**	*Clostridium cadaveris* IFB3C5	CP076620
**09**	*Clostridium amylolyticum* DSM 21864^{T}	FQZO00000000
**10**	*Clostridium fallax* DSM 2631^{T}	FQVM00000000
**11**	*Clostridium intestinale* PC17	CP121166
**12**	*Clostridium polyendosporum* JCM 30710^{T}	BOPZ00000000
**13**	*Clostridium akagii* DSM 12554^{T}	JMLK00000000
**14**	*Clostridium acidisoli* DSM 12555^{T}	FWXH00000000
**15**	*Clostridium subterminale* JCM 1417^{T}	BAAACI000000000
**16**	*Clostridium sulfidigenes* 113A	JPMD00000000
**17**	*Clostridium thiosulfatireducens* src6	OAOI00000000
**18**	*Oxobacter pfennigii* DSM 3222^{T}	LKET00000000
**19**	*Fervidicella metallireducens* AeB^{T}	AZQP00000000
**20**	*Fonticella tunisiensis* DSM 24455^{T}	SOAZ00000000
**21**	*Caloramator fervidus* DSM 5463^{T}	FNUK00000000
**22**	*Caloramator proteoclasticus* DSM 10124^{T}	FQVG00000000
**23**	*Thermobrachium celere* DSM 8682^{T}	CAVN000000000

Table S3. General genomic information and features of strain QWL-01^T and closely related taxa in the genera *Proteiniclasticum* (*Prot.*) and *Youngiibacter* (*Young.*). These detailed data were obtained from the JGI/IMG MER database.

Genome Name	QWL-01^T	*Prot. aestuarii* SCR006^T	*Prot. sediminis* BAD-10^T	*Prot. ruminis* DSM 24773^T	*Young. fragilis* 232.1^T	*Young. multivorans* DSM 6139^T
GenBank ID	CP120965	JAFNJU000000000	JAGSCS000000000	JNKC00000000	AXUN00000000	JAGGKC000000000
Sequencing method	DNBSEQ-T7; MinION	Illumina MiSeq	Illumina HiSeq	Illumina HiSeq	Illumina MiSeq	Illumina NovaSeq S4
Sequencing Status	Finished	Permanent Draft	Permanent Draft	Permanent Draft	Permanent Draft	Permanent Draft
Contigs	1	33	83	29	240	77
Genome size (bp)	3691162	3184189	2985436	3118301	3988233	3670578
GC content (mole%)	50.81	45.57	51.35	43.07	46.57	44.84
Total genes	3478	3134	3027	2939	3783	3587
Protein coding genes	3331	3020	2920	2865	3729	3481
rRNA count	15	14	13	13	4	9
CheckM2 Completeness (%)	99.1	97.52	98.55	96.48	100	100
CheckM2 Contamination (%)	3.72	0.93	0.85	1.68	3.62	1.2
CRISPR repeats	1	0	3	0	1	0
Habitat	Sewage sludge	Tidal flat sediment	Sediment	Yak rumen	Natural gas production-water	Anoxic sludge, oil refinery waste water treatment facility

Table S4. The pairwise analyses of dDDH (upper right part, unit: %) and OrthoANIu (lower left part, unit: %) for strain QWL-01^{T} and other related taxa. [a]Strains: 1. QWL-01^{T}; 2. *Proteiniclasticum aestuarii* SCR006^{T}; 3. *Proteiniclasticum sediminis* BAD-10^{T}; 4. *Proteiniclasticum ruminis* DSM 24773^{T}; 5. *Youngiibacter fragilis* 232.1^{T}; 6. *Youngiibacter multivorans* DSM 6139^{T}; 7. *Clostridium frigidicarnis* DSM 12271^{T}; 8. *Clostridium cadaveris* IFB3C5; 9. *Clostridium amylolyticum* DSM 21864^{T}; 10. *Clostridium fallax* DSM 2631^{T}; 11. *Clostridium intestinale* PC17; 12. *Clostridium polyendosporum* JCM 30710^{T}; 13. *Clostridium akagii* DSM 12554^{T}; 14. *Clostridium acidisoli* DSM 12555^{T}; 15. *Clostridium subterminale* JCM 1417^{T}; 16. *Clostridium sulfidigenes* 113A; 17. *Clostridium thiosulfatireducens* src6; 18. *Oxobacter pfennigii* DSM 3222^{T}; 19. *Fervidicella metallireducens* AeB^{T}; 20. *Fonticella tunisiensis* DSM 24455^{T}; 21. *Caloramator fervidus* DSM 5463^{T}; 22. *Caloramator proteoclasticus* DSM 10124^{T}; 23. *Thermobrachium celere* DSM 8682^{T}.

	1[a]	2	3	4	5	6	7	8	9	10	11	12	13	14	15	16	17	18	19	20	21	22	23
**1**		26.60	30.10	33.00	33.80	36.80	43.90	30.40	36.40	35.80	34.00	37.70	30.50	32.80	37.90	32.10	32.70	27.70	30.80	29.30	25.40	34.00	29.60
**2**	66.28		18.40	18.70	20.80	18.80	32.60	32.00	25.40	37.40	37.60	38.30	28.20	39.90	24.80	32.70	30.50	26.00	25.20	25.70	17.80	35.50	29.30
**3**	68.30	71.57		20.40	28.00	19.20	44.20	38.40	30.10	40.80	36.40	25.30	33.90	39.20	39.30	36.10	35.10	30.70	23.90	32.50	28.40	36.00	29.60
**4**	65.89	73.20	70.96		22.50	20.50	40.30	26.40	28.80	37.50	39.30	41.40	20.30	42.30	28.80	32.60	28.40	22.50	21.70	37.70	32.50	36.90	33.80
**5**	66.81	68.18	68.78	67.56		28.80	42.10	34.60	40.20	23.30	39.30	34.00	26.30	25.70	40.70	36.50	31.60	27.90	33.40	32.60	28.60	36.30	28.70
**6**	65.95	68.45	67.94	67.81	85.22		41.10	34.40	38.90	24.70	39.30	34.30	26.20	12.50	40.40	37.00	34.80	32.70	35.00	34.00	29.70	35.40	30.50
**7**	65.58	66.83	65.70	66.55	66.53	66.82		19.30	21.50	18.70	19.10	19.90	22.10	19.50	21.80	21.10	20.20	23.50	17.30	16.40	16.70	18.20	17.40
**8**	69.47	67.93	67.32	67.43	67.30	67.37	72.22		22.00	20.80	25.10	23.70	20.50	21.30	21.40	23.00	21.10	27.00	23.10	33.40	20.60	21.80	25.00
**9**	66.20	67.09	66.50	66.65	66.83	66.65	70.86	70.37		19.40	21.20	20.70	20.60	19.10	24.90	23.40	21.30	28.70	21.30	25.40	18.50	18.80	20.30
**10**	67.37	67.65	68.58	66.99	67.92	67.75	72.32	71.73	71.62		20.20	19.90	19.70	19.50	18.80	19.70	18.70	29.10	20.80	31.00	18.50	18.50	20.90
**11**	71.11	67.86	67.32	66.48	66.44	66.85	70.64	70.88	70.36	72.34		22.70	20.20	20.60	22.00	22.90	21.80	25.90	12.80	27.70	19.60	22.20	25.30
**12**	65.58	67.08	65.88	66.16	66.42	66.78	71.45	70.45	70.98	73.30	71.66		20.60	20.30	21.70	23.30	21.10	28.70	22.10	24.30	21.40	18.00	20.20
**13**	66.45	66.67	66.19	65.83	66.20	66.69	70.05	69.06	69.27	70.31	69.54	69.99		21.50	18.60	20.00	18.70	29.50	20.80	36.20	27.80	17.40	21.90
**14**	66.93	67.74	67.26	66.18	66.37	67.66	70.37	69.69	70.03	70.75	70.23	70.70	77.23		19.70	19.50	18.70	27.50	28.90	26.90	18.90	19.20	24.30
**15**	64.47	66.84	65.70	66.14	65.94	66.25	72.13	70.27	70.37	70.49	69.90	70.07	69.54	69.99		44.00	42.60	25.30	19.20	32.40	16.80	16.80	19.40
**16**	68.01	67.52	67.99	66.23	66.96	66.76	71.46	70.18	70.46	70.98	70.25	69.65	69.37	69.89	91.23		74.60	25.70	18.90	30.50	17.60	19.20	20.70
**17**	66.96	66.83	66.82	65.98	66.72	66.54	71.16	69.67	69.89	70.51	69.84	69.26	68.81	69.50	90.78	96.99		23.90	19.10	26.10	16.70	16.60	20.50
**18**	65.14	64.90	65.08	64.87	65.38	65.69	67.15	67.10	67.26	67.24	66.71	67.12	66.99	67.46	66.72	67.15	66.26		28.10	20.90	18.70	19.80	28.20
**19**	65.63	66.23	66.05	65.46	65.47	66.11	68.85	68.50	68.35	69.11	68.34	68.75	68.48	69.07	68.79	68.49	68.02	67.80		21.50	18.70	23.20	20.20
**20**	65.68	65.72	66.22	65.05	66.20	65.93	67.66	67.34	68.32	68.19	67.70	68.44	67.40	67.89	67.06	67.06	66.79	67.73	70.74		19.30	22.20	21.20
**21**	65.10	66.64	65.93	65.35	65.53	66.21	68.99	68.60	68.27	69.40	68.11	68.51	68.09	68.63	68.33	68.36	67.96	67.81	71.24	70.29		18.70	20.00
**22**	65.13	66.21	65.66	66.29	65.94	65.86	68.90	68.52	68.58	69.21	68.69	69.33	68.23	68.42	68.60	68.28	68.01	67.71	72.48	70.25	72.16		24.60
**23**	69.92	69.92	67.21	66.18	66.48	66.55	69.14	69.07	68.49	70.25	68.98	68.77	68.44	68.29	68.30	68.63	68.31	67.69	71.33	70.22	72.81	81.43	

**Color bar:**	0	20	40	60	80	100

Table S5. The pairwise analyses of AAI (lower left part, unit: %) for strain QWL-01^T and other related taxa.

[a]Strains: 1. QWL-01^T; 2. *Proteiniclasticum aestuarii* SCR006^T; 3. *Proteiniclasticum sediminis* BAD-10^T; 4. *Proteiniclasticum ruminis* DSM 24773^T; 5. *Youngiibacter fragilis* 232.1^T; 6. *Youngiibacter multivorans* DSM 6139^T; 7. *Clostridium frigidicarnis* DSM 12271^T; 8. *Clostridium cadaveris* IFB3C5; 9. *Clostridium amylolyticum* DSM 21864^T; 10. *Clostridium fallax* DSM 2631^T; 11. *Clostridium intestinale* PC17; 12. *Clostridium polyendosporum* JCM 30710^T; 13. *Clostridium akagii* DSM 12554^T; 14. *Clostridium acidisoli* DSM 12555^T; 15. *Clostridium subterminale* JCM 1417^T; 16. *Clostridium sulfidigenes* 113A; 17. *Clostridium thiosulfatireducens* src6; 18. *Oxobacter pfennigii* DSM 3222^T; 19. *Fervidicella metallireducens* AeB^T; 20. *Fonticella tunisiensis* DSM 24455^T; 21. *Caloramator fervidus* DSM 5463^T; 22. *Caloramator proteoclasticus* DSM 10124^T; 23. *Thermobrachium celere* DSM 8682^T.

	**1[a]**	**2**	**3**	**4**	**5**	**6**	**7**	**8**	**9**	**10**	**11**	**12**	**13**	**14**	**15**	**16**	**17**	**18**	**19**	**20**	**21**	**22**	**23**
**1**																							
**2**	49.27																						
**3**	50.87	66.88																					
**4**	49.29	70.59	65.46																				
**5**	51.58	57.54	58.47	56.65																			
**6**	50.64	57.74	58.12	56.95	89.68																		
**7**	45.99	48.07	47.96	47.74	47.65	48.12																	
**8**	46.05	47.98	48.04	47.89	48.11	48.22	57.80																
**9**	46.59	48.77	49.04	48.77	49.01	48.96	56.19	54.86															
**10**	46.29	48.55	48.36	48.56	48.69	49.21	58.21	55.95	58.40														
**11**	45.15	47.85	47.76	47.54	48.39	48.86	54.29	53.48	55.55	57.70													
**12**	46.03	48.35	48.18	48.43	49.09	49.02	55.82	54.64	57.35	61.95	59.43												
**13**	44.23	45.96	45.98	45.44	46.28	46.20	52.11	50.54	52.17	52.67	52.99	54.75											
**14**	44.48	46.57	46.15	45.98	46.78	46.98	53.18	51.49	52.93	53.67	53.50	55.93	72.33										
**15**	45.39	47.49	46.68	47.27	46.82	47.45	55.12	53.62	53.59	53.55	51.62	52.35	50.60	51.88									
**16**	46.06	47.90	47.28	47.71	47.38	47.76	55.08	54.01	53.99	53.75	52.00	52.85	51.01	52.24	90.37								
**17**	45.84	47.34	46.81	47.06	46.64	46.84	54.95	53.18	52.85	54.10	50.92	52.41	50.25	51.20	87.10	93.78							
**18**	43.81	44.41	46.25	44.41	45.43	45.64	47.00	47.02	48.01	47.64	48.13	49.93	47.40	48.23	46.79	47.09	47.17						
**19**	44.69	46.48	46.25	46.18	46.53	46.70	49.80	49.23	51.00	50.14	49.04	51.01	48.78	49.62	50.20	49.86	50.09	50.75					
**20**	44.55	46.62	46.79	46.07	47.53	46.97	49.88	49.17	51.31	50.46	50.57	52.06	49.94	50.86	48.79	49.02	49.39	51.53	60.09				
**21**	45.71	47.28	46.42	47.14	47.06	47.41	50.88	49.84	51.06	51.15	49.91	50.80	49.19	50.03	49.87	50.37	51.34	51.39	60.74	59.81			
**22**	44.95	46.78	46.49	46.78	46.86	47.37	50.01	49.23	50.78	50.70	49.36	50.85	49.14	49.32	49.60	49.97	50.12	50.59	60.89	59.15	63.10		
**23**	45.42	47.00	46.72	46.86	47.04	47.47	50.64	50.10	51.24	51.17	49.91	51.19	49.14	49.85	50.32	50.32	50.96	50.85	60.54	59.55	64.35	82.98	

Color bar: 0 20 40 60 80 100

Table S6. Genome-based inference of the cell envelope structure of strain QWL-01^T.

Functional category	Representative genes/proteins (accession numbers)	Presence in genome	Interpretation
Lipid A biosynthesis (LPS)	*lpxA, lpxC, lpxD, lpxB, lpxK*	Not detected	Core pathway for LPS absent
Kdo biosynthesis (LPS core)	*kdsA, kdsB*	Not detected	No evidence for LPS core formation
LPS transport system	*lptA, lptB, lptC, lptD, lptE, lptF, lptG*	Not detected	LPS translocation machinery absent
Lipid A flippase	*msbA*	Not detected	Essential for diderm outer membrane absent
Outer membrane β-barrel assembly	*bamA* (OMP85 family)	Not detected	No outer membrane protein assembly system
Outer membrane lipoprotein trafficking	*lolC, lolD, lolE, lolA, lolB*	Not detected	Incompatible with canonical diderm envelope
Wall teichoic acid biosynthesis (WTA)	*tagA, tagB, tagD, tagF*	Not detected	Not uncommon in Clostridiaceae
Transferring D-alanine to lipoteichoic acid (LTA)	*dltB* (WFF74486.1; WFF73734.1; WFF71398.1;	Detected	The key gene to modify Gram-positive bacterial cell walls
Sortase system	*srtA* (WFF73011.1)<br>*srtB* (WFF72497.1)<br>*srtC* (WFF71931.1)	Detected	Typical of monoderm Gram-positive bacteria
Cell wall-anchored proteins	LPXTG motif proteins (WFF71933.1; WFF73041.1; WFF72499.1)	Detected	Strong evidence for peptidoglycan anchoring

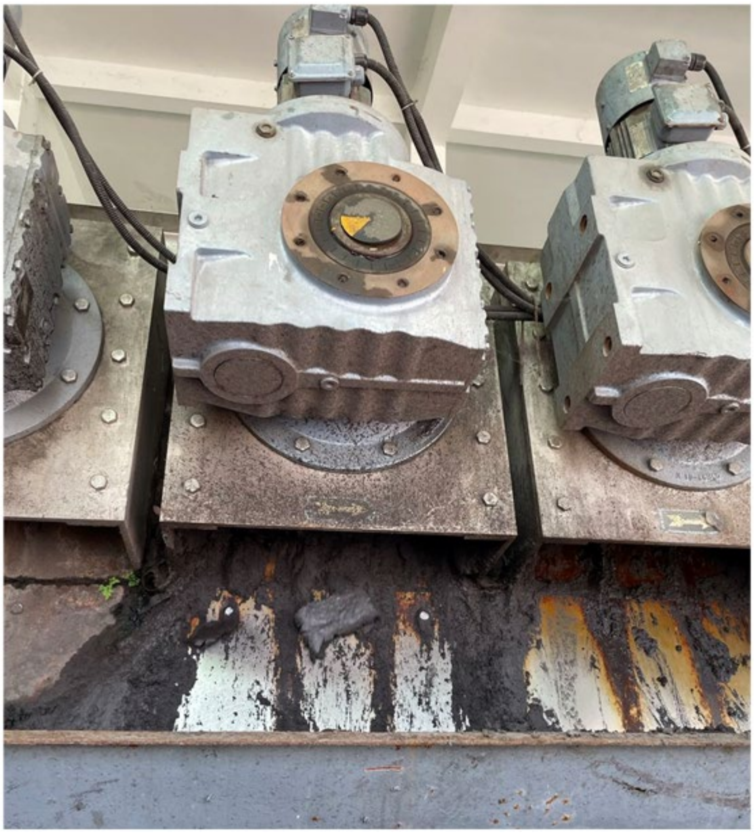

Figure S1. A photograph of the sludge dewatering devices and dehydrated sludge collected at the wastewater treatment plant of the Sanming Steel Co. Ltd. in Sanming City, Fujian Province, China, taken on June 25, 2021.

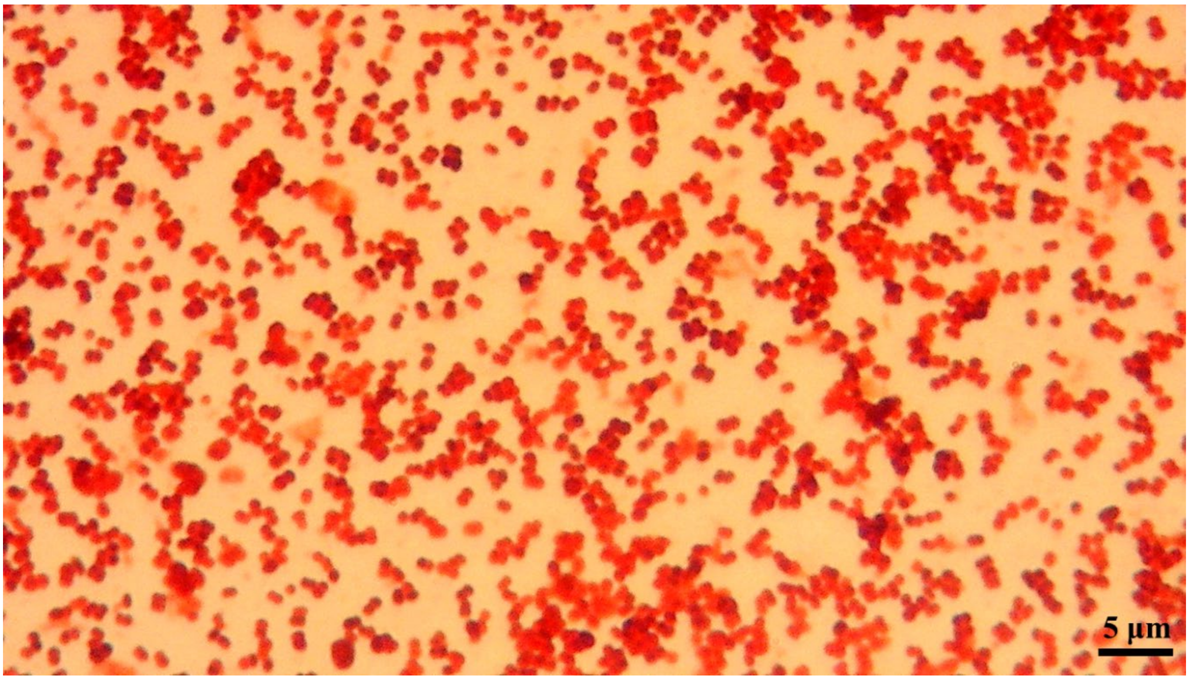

Figure S2. Gram-staining micrograph of strain QWL-01^{T}. Cells were stained using the standard Gram-staining procedure and observed under a light microscope. Strain QWL-01^{T} exhibited a Gram-negative staining reaction, appearing red after counterstaining. Scale bar, 5 µm.

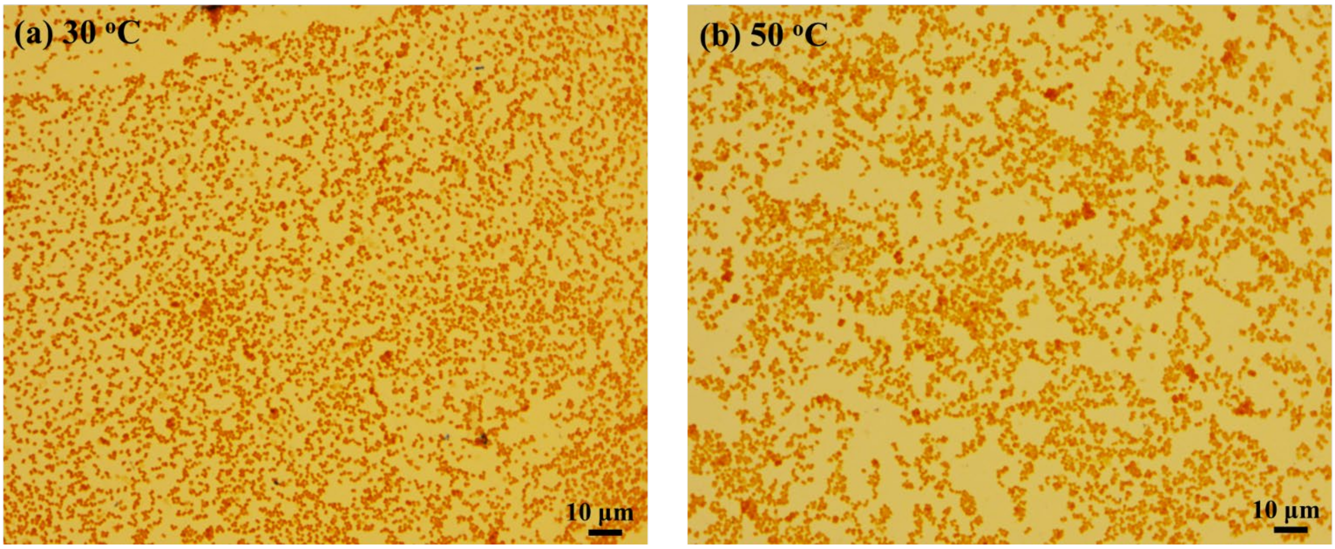

Figure S3. Malachite green spore-staining micrographs of strain QWL-01^T. (a) Cells cultured anaerobically at 30 °C for 3 days and then stained using the malachite green spore-staining method. (b) Cells initially cultured at 30 °C for 16 h and subsequently incubated at 50 °C for 1 day prior to malachite green staining. In both conditions, strain QWL-01^T exhibited no detectable endospore formation, with vegetative cells counterstained red and no green-stained spores observed. Scale bar, 10 μm

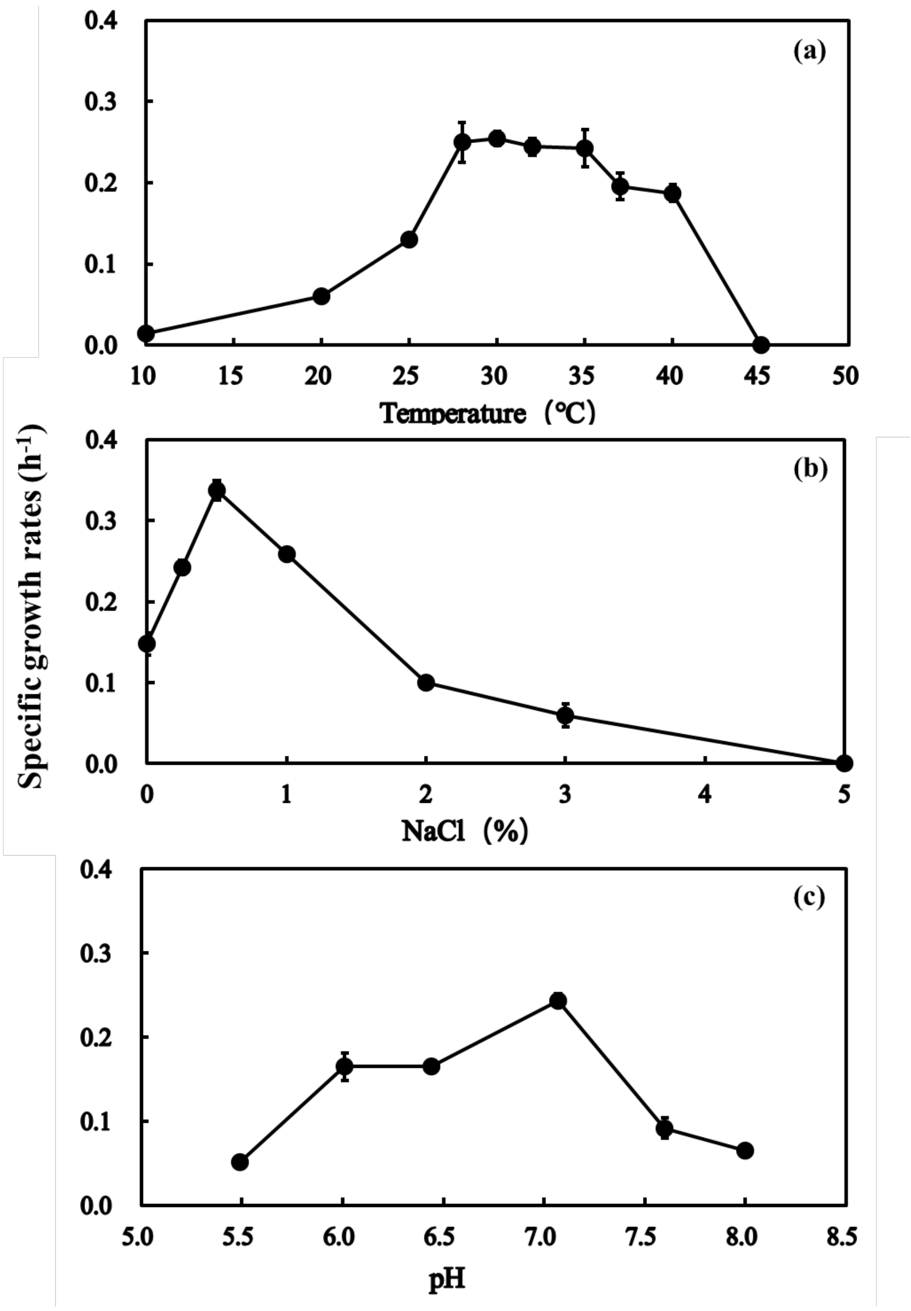

Figure S4. Influence of (a) temperature, (b) NaCl concentration and (c) pH on the growth of strain QWL-01^T inoculated into modified DSM 120 media. The growth temperature tests were conducted at 4 °C, 10 °C, 20 °C, 25 °C, 28 °C, 30 °C, 32 °C, 35 °C, 37 °C, 40 °C, and 45 °C in the presence of 0.25% NaCl. The growth experiment of NaCl concentrations were set at 0 %, 0.25%, 0.5%, 1%, 2%, 3%, and 5%. The pH values for growth tests were conducted at 5.5, 6.0, 6.4, 7.1, 7.6, and 8.0. The temperature was set to 30 °C for the NaCl concentration and pH growth tests. Specific growth rates were calculated based on the increase in optical density during the logarithmic growth phase and were presented as the means of triplicate or quadruplicate cultures.

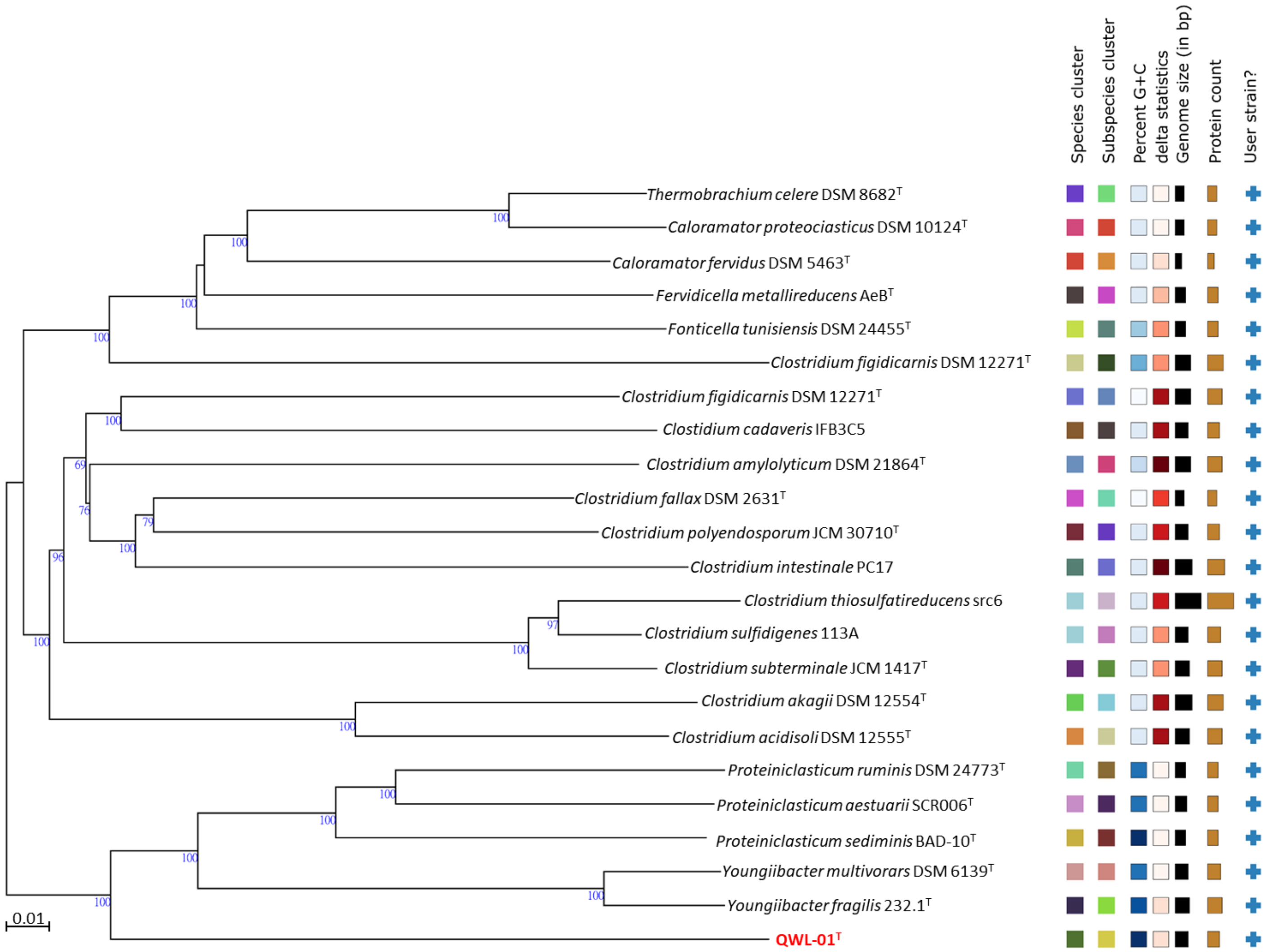

Figure S5. TYGS phylogenetic tree based on whole-proteome showing relationships between strain QWL-01^{T} (red) and related taxa (listed in Table S2). Tree inferred with FastME 2.1.4 from Genome BLAST Distance Phylogeny (GBDP) distances calculated from genome sequences. Branch lengths are scaled in terms of GBDP distance formula d5; numbers at the nodes are GBDP pseudo-bootstrap support values from 100 replicates. Leaf labels are annotated by affiliation to species and subspecies clusters, genomic G+C content, δ values, overall genome size, and number of proteins.
